# The role of *let-7b* in the inhibition of hepatic stellate cell activation by rSjP40

**DOI:** 10.1371/journal.pntd.0009472

**Published:** 2021-06-23

**Authors:** Xiaolei Sun, Li Zhang, Yuting Jiang, Aihong Li, Dandan Zhu, Jiangrong Wu, Yinong Duan

**Affiliations:** 1 Department of Pathogen Biology, School of Medicine, Nantong University, Jiangsu, People’s Republic of China; 2 Department of Clinical laboratory, Huangshi Central Hospital, Affiliated Hospital of Hubei Polytechnic University, Edong Healthcare Group, Hubei, People’s Republic of China; 3 Department of Neurology, Affiliated Hospital of Nantong University, Jiangsu, People’s Republic of China; University of the District of Columbia, George Washington University School of Medicine and Health Sciences, UNITED STATES

## Abstract

**Background:**

Hepatic stellate cells (HSCs) are one of the main cell types involved in liver fibrosis induced by many factors, including schistosomes. Previous studies in our lab have shown that recombinant P40 protein from *Schistosoma japonicum* (rSjP40) can inhibit HSC activation *in vitro*. Let-7b is a member of the let-7 microRNA family and plays an inhibitory role in a variety of diseases and inflammatory conditions. In this study, we investigated the role of let-7b in the inhibition of HSC activation by rSjP40.

**Methods:**

Expression of let-7b was detected by quantitative real-time PCR. A dual luciferase assay was used to confirm direct interaction between let-7b and collagen I. We also used western blot to assess protein levels of TGFβRI and collagen type I α1 (COL1A1).

**Results:**

We found that rSjP40 up-regulates expression of let-7b in HSCs. Let-7b inhibits collagen I expression by directly targeting the 3’UTR region of the collagen I gene. Furthermore, we discovered that let-7b inhibitor partially restores the loss of collagen I expression caused by rSjP40.

**Conclusion:**

Our research clarifies the role of let-7b in the inhibition of HSC activation by rSjP40 and will provide new insights and ideas for the inhibition of HSC activation and treatment of liver fibrosis.

## Author summary

Hepatic stellate cells (HSCs) are one of the main cell types involved in liver fibrosis induced by schistosomes. Our previous studies have shown that recombinant P40 protein from *Schistosoma japonicum* (rSjP40) can inhibit HSC activation *in vitro*. In this study, we found that rSjP40 up-regulates expression of let-7b in HSCs. Let-7b is a member of the let-7 microRNA family and plays an inhibitory role in a variety of diseases and inflammatory conditions. Let-7b inhibits collagen I expression by directly targeting the 3’UTR region of the collagen I gene. Furthermore, we discovered that let-7b inhibitor partially restores the loss of collagen I expression caused by rSjP40. Our research clarifies the role of let-7b in the inhibition of HSC activation by rSjP40 and will provide new insights and ideas for the inhibition of HSC activation and treatment of liver fibrosis.

## Introduction

Liver fibrosis is a response to chronic liver damage caused by various factors, such as schistosomiasis, viral hepatitis, alcoholism and cholestasis [1,2]. Hepatic stellate cells (HSCs) are considered to be one of the main cell types involved in liver fibrosis [3]. During the process of liver fibrosis, HSCs are activated and differentiate into myofibroblasts. Subsequently, activated HSCs primarily produce collagen, α-smooth muscle actin (α-SMA) and matrix metalloproteinase [4].

Schistosomiasis is a chronic disease and a serious social health problem worldwide [5]. However, studies have shown that the eggs of both *Schistosoma japonicum* and *Schistosoma mansoni* can inhibit HSC activation and fibrogenesis [6,7]. Previous studies in our lab have also shown that soluble egg antigens (SEA) from *Schistosoma japonicum* could suppress activation and proliferation of HSCs [8]. SjP40 is the main component of *Schistosoma japonicum* SEA [9]. We found that recombinant SjP40 (rSjP40) can also inhibit HSC activation caused by TGF-β1 [10]. We also confirmed that rSjP40 promotes cellular senescence via STAT3/p53/p21 and SKP2/P27 pathways [11,12].

MicroRNAs (miRNAs) represent a class of non-coding RNAs that negatively regulate expression of target genes at the post-transcriptional level by binding to the 3’UTR regions of target genes [13,14]. MiRNAs have been implicated in the process of fibrosis via various signaling pathways. For example, miR-219 can modulate liver fibrosis by directly targeting tumor growth factor β receptor 2 (TGFBR2) [15], while miR-34a-5p targets Smad4 and modulates TGF-β1/Smad3 pathway in HSCs to ameliorate liver fibrosis [16]. Lethal-7 (let-7) was one of the earliest miRNAs to be discovered [17]. Let-7b is a member of the let-7 family and plays an important role in the development of many liver diseases, including viral infection [18,19], alcoholic liver injury [20], and hepatocellular carcinoma [21,22]. Recently, let-7b has been reported to inhibit liver fibrosis in *Schistosoma japonicum*-infected mice by reducing expression of TβRI [23]. Therefore, we wondered whether let-7b was related to the inhibition of HSC activation caused by rSjP40. In our study, we found that let-7b is up-regulated under stimulation by rSjP40 and is involved in the inhibition of rSjP40-induced HSC activation through direct targeting of collagen I.

## Material and methods

### Cell culture

The LX-2 human HSC line was obtained from Nantong Third People’s Hospital. LX-2 cells were cultured in Dulbecco’s modified Eagle’s Medium (DMEM, Thermo Fisher, USA) with 10% fetal bovine serum (FBS) in a 37°C, 5% CO_2_ incubator. LX-2 cells were inoculated into 6-well culture plates or 24 well plates for 12 h and then stimulated with 20μg/mL of rSjP40. rSjP40 protein was obtained as previously described [10].

### Transfection of miRNA mimic and inhibitor

The let-7b mimic, let-7b inhibitor and respective negative controls were compounded by GenePharma (China). All constructs were transiently transfected into cells using the FuGENE transfection reagent (Promega, USA) according to the manufacturer’s instructions. Cells were harvested for further experimental analysis 48h after transfection.

### Western blot

Total protein was extracted from LX-2 cells on ice using standard methods. Protein was quantified by Bradford method (Sangon, China). Each protein sample was separated by 10% SDS-PAGE, transferred onto a PVDF membrane (Merck, Germany) and blocked with 5% non-fat milk at room temperature for 2 hours. The membrane was then incubated with mouse anti-collagen I antibody (Abcam, USA) or rabbit anti-TGFβR I antibody (Santa Cruz, USA) overnight at 4°C, then with secondary antibodies (Santa Cruz, USA) for 1h at room temperature. Finally, protein bands were detected by a chemiluminescence (ECL) kit (Merck, Germany) and quantified using Image Lab (Bio-Rad, USA). For the above experiments, GAPDH was used as an internal control.

### RNA extraction and Quantitative real-time PCR (RT-qPCR)

miRNA was isolated using RNAiso for Small RNA (TAKARA, Japan) and reverse-transcribed into cDNA using the Mir-X^TM^ miRNA First-Strand Synthesis Kit (TAKARA, Japan). RT-qPCR analysis was performed using the SYBR Premix Ex Taq kit (TAKARA, Japan) and a StepOnePlus real-time PCR system (Applied Biosystems, USA). U6 was used as an internal control. All samples were run in triplicate.

### Dual-luciferase reporter assay

The sequences of the let-7b promoter and the 3′UTR of collagen I were obtained from the National Center for Biotechnology Information (NCBI) website. TargetScan was used to predict binding sites of let-7b and collagen I. A fragment of the let-7b promoter was cloned into a pGL3-basic vector (Promega, USA) and designated as pGL3-pro-let-7b. Wild-type or mutated sequences from the 3’UTR of collagen I were cloned into a psiCHECK-2 luciferase vector (Promega, USA). These reporter plasmids were respectively transfected into LX-2 cells. After transfection for 12 hours, 20 μg/mL of rSjP40 was added to stimulate the cells for an additional 48 hours. Luciferase activity was detected on a luminometer according to the instructions of the dual-luciferase reporter assay system (Promega, USA).

### Statistical analysis

Each of the above experiments was repeated three times for statistical analysis. All data are expressed as mean ±SEM, and differences between groups were analyzed by one-way ANOVA. When *p* < 0.05, a difference was considered statistically significant. Adobe Photoshop 7.0 and SPSS 15.0 software were used for plotting and analysis of statistical data.

## Results

### Let-7b is highly expressed in rSjP40-treated LX-2 cells

**Fig 1:**
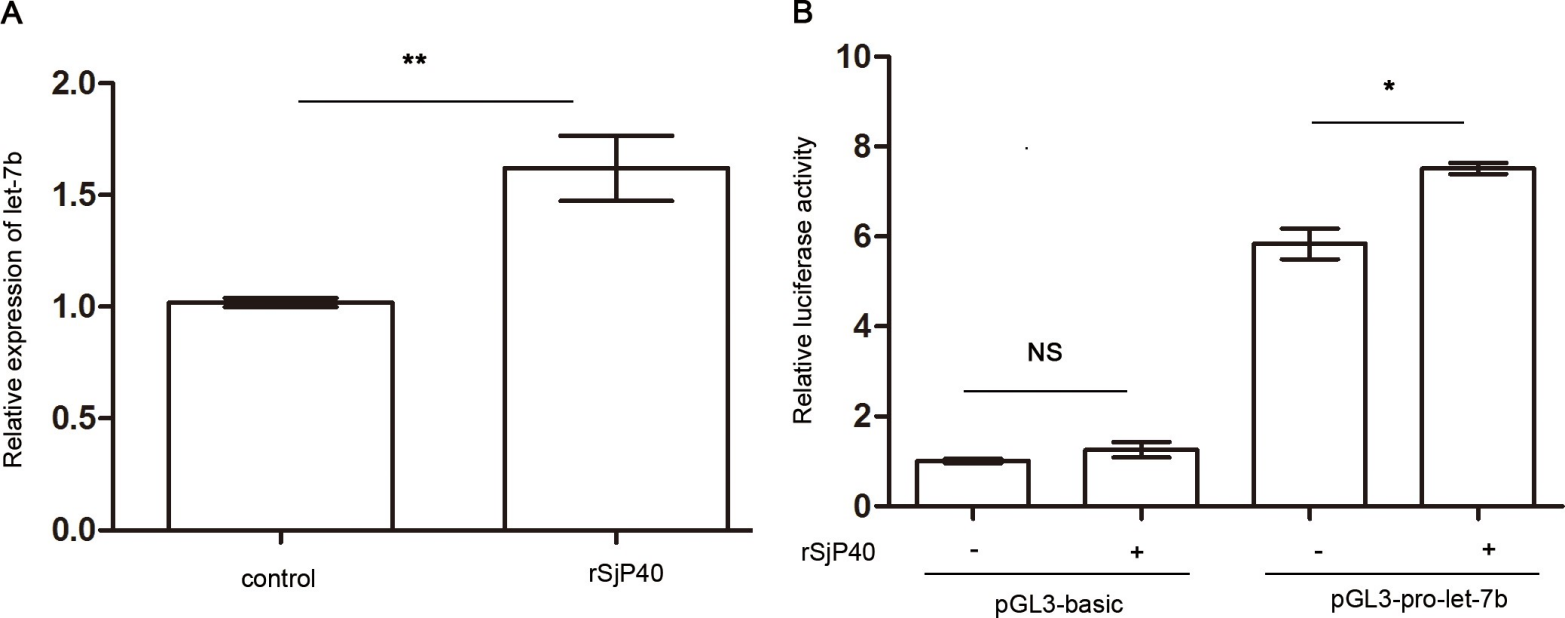
Expression of let-7b in LX-2 cells under rSjP40 stimulation. (A): Let-7b is highly expressed in rSjP40-treated LX-2 cells. (B): rSjP40 increases promoter activity of pGL3-pro-let-7b. *p<0.05, **p<0.01, NS, *p*>0.05, compared with the control group. Data were taken as mean ± SEM of at least three independent experiments.

We first used RT-qPCR to examine expression of let-7b in LX-2 cells treated with rSjP40. This analysis determined that expression of let-7b increased significantly after rSjP40 stimulation *in vitro* (Fig 1A). Meanwhile, dual-luciferase reporter gene results showed that rSjP40 increased promoter activity of pGL3-pro-let-7b but had no effect on the pGL3-basic group (Fig 1B). This result is similar to those of Tang et al. [23], as they found that let-7b was down-regulated in *S*. *japonicum-*infected liver tissues, and we found that rSjP40 can inhibit HSC activation *in vitro* [10], leading us to suspect that rSjP40 can restrain activation of HSCs by up-regulating let-7b expression.

### Let-7b targets and degrades collagen I and TGFβI in LX-2 cells

**Fig 2:**
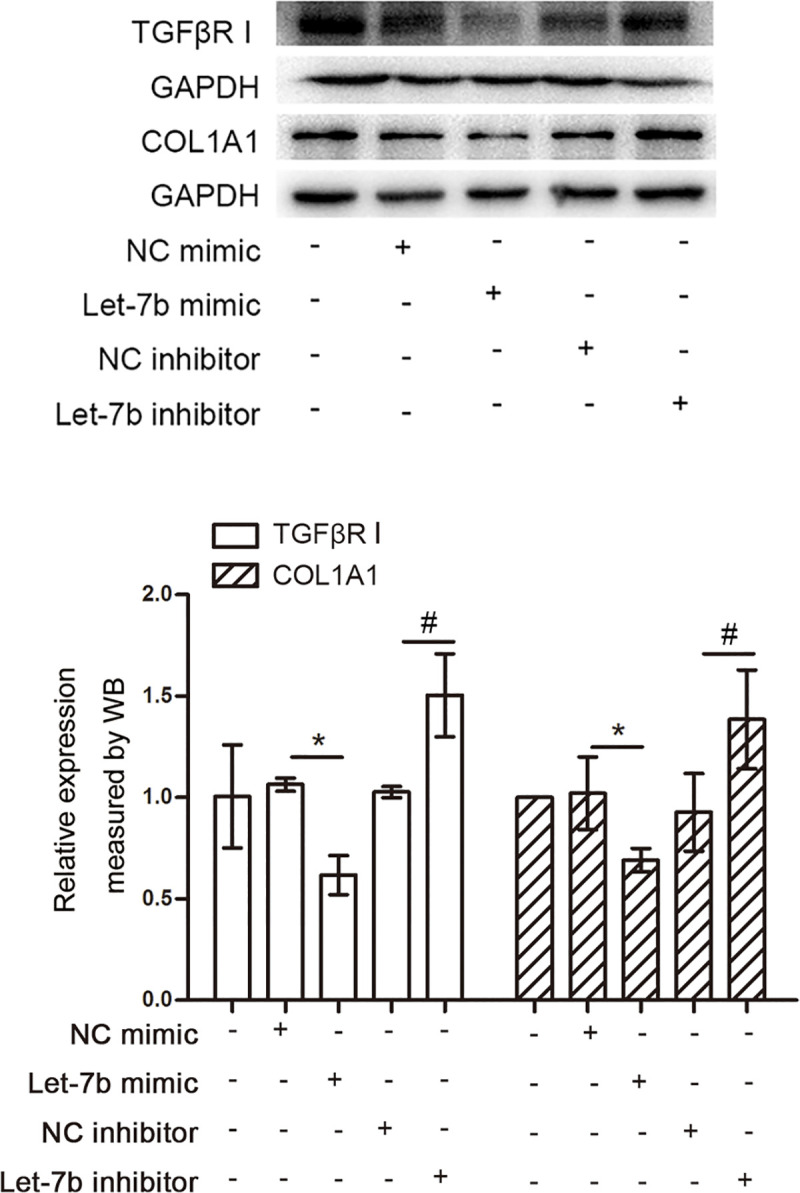
Collagen I and TGFβI are targets of let-7b. Results of western blot show that the let-7b mimic down-regulates expression of collagen I and TGFβI, while the let-7b inhibitor up-regulates expression of collagen I and TGFβI in LX-2 cells. *p<0.05, compared with group transfected with NC mimic. ^#^*p*<0.05, compared with group transfected with NC inhibitor.

Studies have shown that let-7b is negatively correlated with collagen I and TGFβI [24,25,26,27], leading us to wonder whether collagen I and TGFβI were target genes of let-7b. We transfected either a let-7b mimic or inhibitor into LX-2 cells. The results of western blot (Fig 2) demonstrate that collagen I protein expression was suppressed in LX-2 cells transfected with the let-7b mimic, while collagen I protein expression was enhanced in LX-2 cells transfected with the let-7b inhibitor. Similar changes to TGFβI expression were observed in LX-2 cells transfected with the let-7b mimic or inhibitor (Fig 2).

### rSjP40 inhibits LX-2 cell activation by the let-7b/collagen I pathway

**Fig 3:**
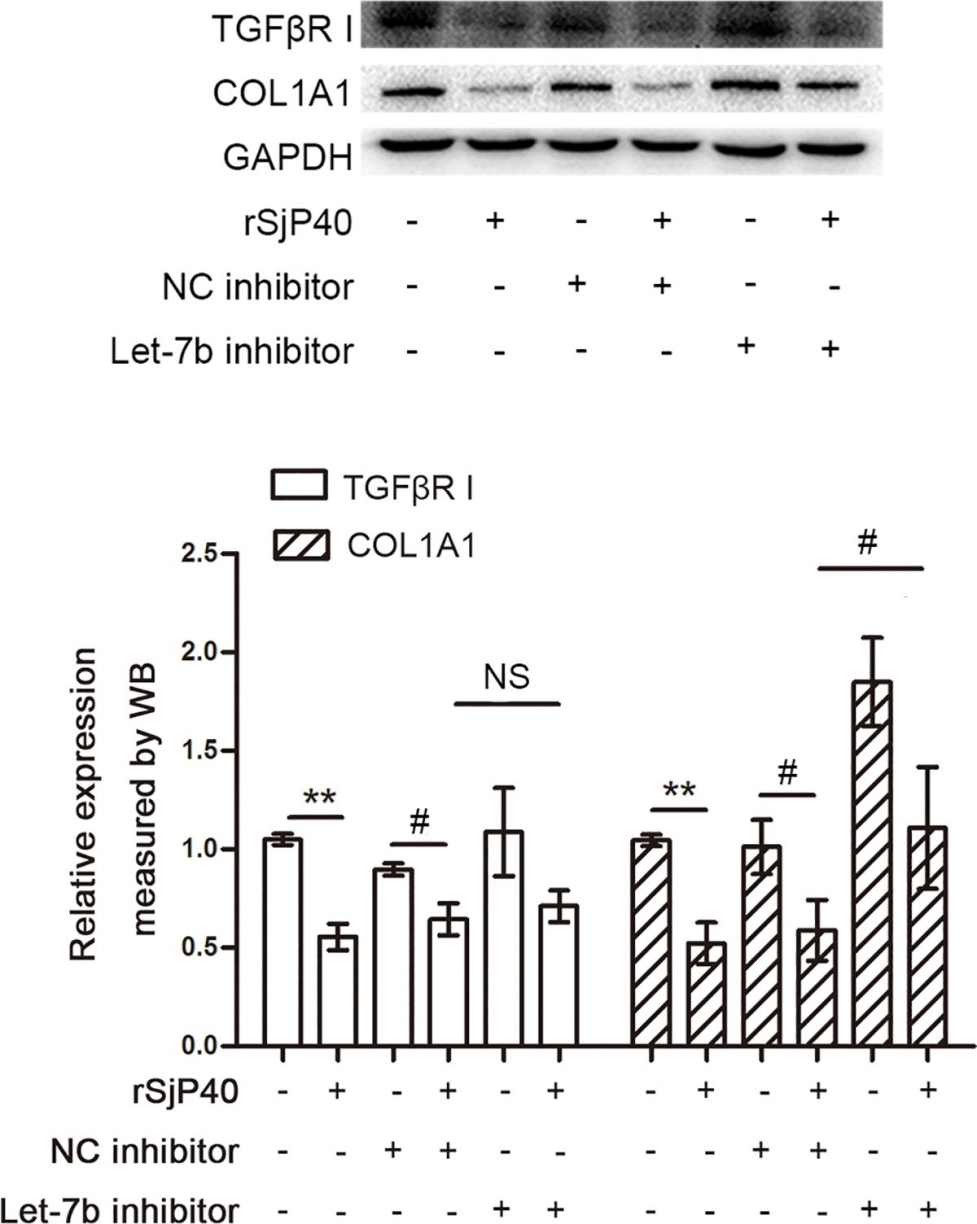
Let-7b participates in rSjP40-induced inhibition of LX-2 cell activation by targeting collagen I. rSjP40 suppresses levels of collagen I and TGFβI, but only expression of collagen I could be restored by the let-7b inhibitor. **p<0.01, compared with control group. ^#^*p*<0.05, compared with group transfected with NC inhibitor and treated with rSjP40. ^NS^*p*>0.05, compared with group transfected with NC inhibitor and treated with rSjP40.

We further explored whether let-7b could be involved in rSjP40-induced inhibition of HSC activation. Results of western blot showed that expression of collagen I protein was decreased significantly by rSjP40 stimulation in LX-2 cells (Fig 3). Compared with the NC group, higher collagen I protein expression was observed in the group transfected with let-7b inhibitor. In addition, collagen I protein levels in the rSjP40 and let-7b inhibitor groups were partially increased compared with those of the rSjP40 and NC groups. However, the let-7b inhibitor did not restore expression of TGFβI under rSjP40 stimulation (Fig 3). All results indicate that the let-7b inhibitor partially restores the reduced collagen I expression caused by rSjP40. It is well known that collagen acts as a fibrotic factor [4,28], so the above data may suggest that let-7b participates in inhibiting HSC activation by rSjP40 and improves fibrosis by acting directly on collagen I.

### Let-7b can bind directly to the 3’UTR of collagen I in LX-2 cells

**Fig 4:**
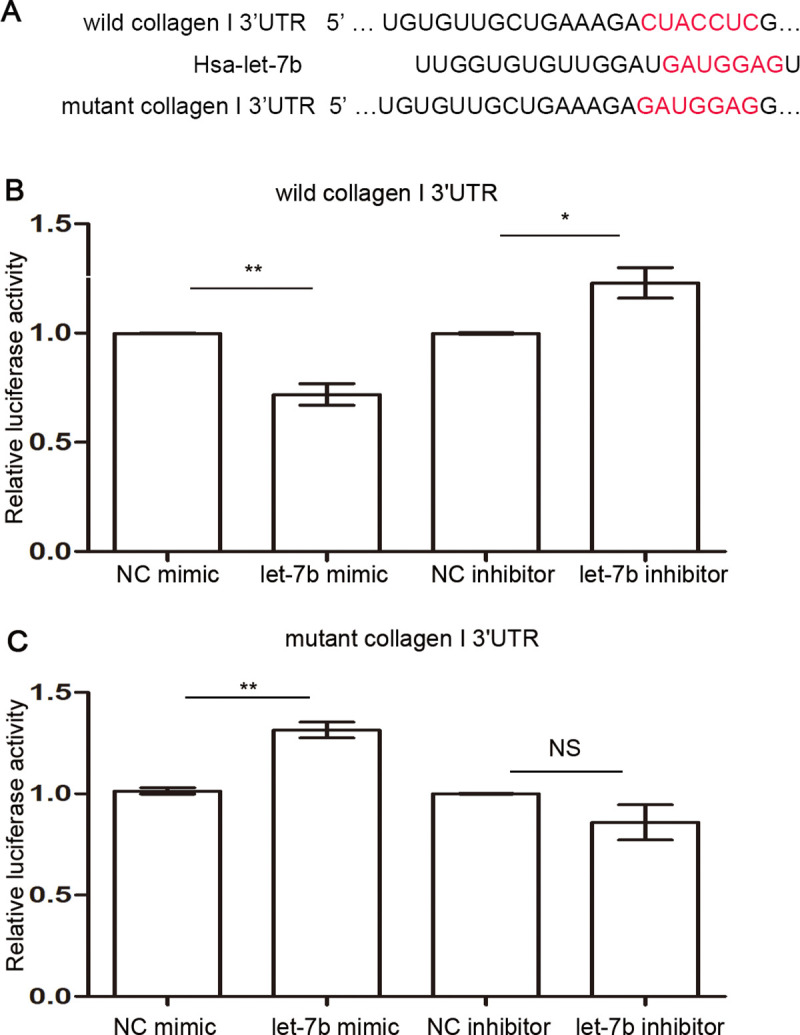
Let-7b can directly bind the 3’UTR of collagen I in LX-2 cells. (A) Binding sites of let-7b and collagen I as predicted by TargetScan. (B-C) Dual luciferase reporter gene assay showing that let-7b can directly bind the 3′UTR of collagen I. *p<0.05, **p<0.01, NS, *p*>0.05, compared with the control group. Data were taken as mean ± SEM of at least three independent experiments.

We found that collagen I has a let-7b binding site predicted by TargetScan (Fig 4A). To further verify that let-7b could directly bind to collagen I, we successfully constructed wild-type and mutant plasmids containing the collagen I-3’UTR. Dual-luciferase reporter gene results indicated that fluorescence activity was decreased in cells transfected with let-7b mimic and wild type collagen I, while fluorescence activity was increased in the let-7b inhibitor-treated group (Fig 4B). At the same time, we co-transfected the mutated plasmid with let-7b mimic or inhibitor. We found that the changes to luciferase activity seen in wild-type plasmid transfected cells disappeared in mutant-type transfected cells (Fig 4C). All of the above results indicate that let-7b can adjust expression of collagen I through directly binding the collagen I 3′UTR.

## Discussion

HSC activation is important to the development of liver fibrosis [29,30]. Once activated, HSCs secrete inflammatory cytokines and up-regulate the expression of fibrotic markers, including α-SMA and collagen, which are key proteins leading to liver fibrosis [31]. Notably, attenuation of the source of liver injury could revert HSCs to an inactivated state or induce apoptosis [32,33]. Therefore, reversal of HSC activation is the key to controlling progression of liver fibrosis [34]. Schistosomiasis is a transmitted disease caused by helminth worms, and *Schistosoma japonicum* is the main human schistosome in China [5]. *Schistosoma japonicum* infection usually causes chronic damage and liver fibrosis, and it may eventually develop into portal hypertension or cirrhosis [35]. It is generally believed that liver fibrosis is the main pathological change of schistosomiasis [36,37]. While conventional view suggests that schistosome eggs are the major inducer of fibrosis, both our results and the results of Anthony et al. demonstrate that some components of schistosome eggs can inhibit hepatic fibrosis under certain conditions [6,7,38,39]. SjP40, which has a molecular weight of 40kDa, is an important component of *Schistosoma japonicum* eggs. It is known as SmP40 in *Schistosoma mansoni* and SjP40 in *Schistosoma japonicum* [40]. Recent studies have shown that rSjP40 restrains expression of fibrogenic markers α-SMA and COL1A1 in HSCs [9]. This indicates that SjP40 may have an inhibitory effect on fibrosis. Hence, the exploration of mechanisms by which rSjP40 could inhibit hepatic fibrosis is a topic of major interest.

Many studies have confirmed that miRNAs play an important role in liver fibrosis. MiR-221 and miR-222 are enhanced in early and late liver fibrosis and have potential use as biomarkers of liver fibrosis [41]. MiR-31 targets FIH1 and activates HSCs during liver fibrosis [42]. MiR-29a and miR-652 improve liver fibrosis through inhibiting differentiation of CD4+ T cells [43]. MiR-338-3p [44], miR-29 [45] and miR-21 [46] are involved in the activation and proliferation of HSCs. In addition, Ye et al. demonstrated that knockdown of miR-145 may directly target adducing-3 to cause liver fibrosis [47]. In our study, we confirmed that let-7b is increased in rSjP40-treated LX-2 cells and may participate in rSjP40-inhibited HSC activation.

With regard to non-coding RNAs, the let-7 miRNA family represents the first known human miRNA family, consisting of let-7a / b / c / d / e / f / g / i and miR-98 [48,49]. Let-7b has been found to be down-regulated in many diseases. Let-7b-5p acts as a tumor suppressor in multiple myeloma, and insulin-like growth factor receptor 1 (IGF1R) is negatively regulated by let-7b-5p at the post-transcriptional level [50]. In renal cell carcinoma, expression of both let-7b and let-7c were significantly decreased, and expression of let-7b was further associated with pathological grade. Cheng et al. observed that let-7b inhibits activity of hepatitis C virus (HCV) replicators and down-regulates accumulation of HCV, thus reducing HCV infectivity [18]. Let-7b could also suppress proliferation of HCC cells by increasing levels of p21 [21]. In our study, we further found that let-7b targets collagen I and TGFβI. However, although rSjP40 downregulates TGFβI expression [10], treatment with a let-7b inhibitor failed to partially restore the reduced TGFβI expression caused by rSjP40. Hence, we hypothesize that other pathways may affect TGFβI expression.

## Conclusion

In conclusion, we observed that expression of let-7b was significantly increased when rSjP40 was added to LX-2 cells and determined that let-7b is involved in the inhibition of HSC activation by directly targeting collagen I. Our study provides new ideas for the use of let-7b miRNA to inhibit HSC activation during liver fibrosis.
